# M. Andral on the Changes of the Blood in Disease

**Published:** 1842-06-25

**Authors:** 


### *M. Andral on the Changes of the Blood in Disease.*

*Sanguineous Temperament.*—According to the researches of MM. Andral and Gavarret it would seem that the common notion that the blood in persons of this temperament contains a larger quantity than usual of its solid or fibrinous constituent is not strictly correct. There is but little increase in the proportion of this element; whereas that of the red globules is generally very considerably augmented—from 127 to 135 or 140 parts in 1000. If we examine the blood of such persons before it coagulates, it is found to be of an extremely bright red colour. The clot is usually large, from the retention of a large quantity of the serum in its meshes; but its consistence is not greater than in health; and often it is even less. One of the chief characters of this blood is that *it very rarely exhibits a perfect buffy coat*—although the very contrary opinion is so generally asserted. This circumstance is owing to the small proportion of the fibrine in comparison with that of the globules, which, as we have said, are predominant.

In persons of the sanguineous temperament, all the functions of the body are usually performed with great activity; life, so to speak, is in excess; and this excess is proportionate to the increase of the red globules. The digestive process goes on rapidly and efficiently; the respiratory apparatus is largely developed; and the minute capillary vessels are always more highly injected than in persons of other temperaments. The heat of the body is high; the perspiration is easy; and there is an abundant secretion of deep-coloured urine, which usually contains a large quantity of saline matter. The energies of the brain are easily exalted; the passions are quick, and vehement; and yet the sensibility is by no means excessive, and never so acute as in persons of the nervous temperament. Hence the class of diseases known by the term *neuroses* is not common in plethoric individuals. It would seem that, as the proportion of the red globules increases, so the sensibility of the system diminishes, and *vice versa.* An extreme and morbid acuteness of the nervous system is well known to be a characteristic symptom of chlorosis and anæmia.

The pathological conditions of most frequent occurrence in plethora are congestion, hæmorrhage, and fever. Genuine inflammation is much less frequent; we might even assert—in opposition indeed to the generally received opinion—that *plethoric persons are not more liable to the phlegmasiæ than those of other temperaments.* From the very circumstance of the globules being in an increased proportion, the normal relation between the proportion of this element and that of the fibrine is disturbed, and the consistence of the blood is rendered actually less than it is in health. Blood-letting, from its marked influence in reducing the proportion of the globules, always speedily relieves the diseases arising from plethora; its influence on the fibrine is much less decided and speedy.

*The Lymphatic Temperament and Anæmia.*—The general fact to be mentioned in reference to this temperament is the diminution of the physical forces, and an enfeebled state of many of the functions of the body. There is usually a strong disposition to the development of scrofulous disease. M. Lecanu has stated, and M. Andral confirms the opinion, that the proportion of the red globules is always low in persons of this temperament. The surface is usually pale and puffy; the iris exhibits a more than ordinary clear hue; and the pilous system is but little developed. When any phlegmasia occurs in scrofulous persons, it proceeds in its course slowly, and less readily to resolution—a termination which is always long of being fairly established, and is apt to be interrupted by a variety of accidents.

The lymphatic temperament, when much exaggerated, leads, as we have already said, to the development of scrofula; the anæmic to that of chlorosis. In spontaneous or idiopathic anæmia, the proportion of the fibrine is often but little diminished; but, in that produced by great hæmorrhages, the blood is always deficient in this element. The proportion of the globules is sometimes remarkably diminished; it has been known to fall from one hundred and twenty-seven—the normal standard—down to forty, and even as low as twenty-seven.

Although all the physical functions suffer, whenever there is any considerable diminution of the red globules the energies of the brain are often surprisingly little affected; the sensory functions are usually exalted; and the activity of the mind is not unfrequently increased. But the digestion is always more or less disturbed, and the patient often suffers exceedingly from one of the most severe and intractable forms of gastrodynia, as well as from headaches and confusion of the sight, and of the other especial sensations. The actions of the heart and lungs, too, are very frequently more or less severely deranged. Every practical physician is well aware how much anæmic patients usually suffer from violent palpitations, and that the tictac of the heart is often accompanied with a blowing sound, such as is heard in cases of contraction of the arterial orifices. The blowing sound may be either constant, or it may be intermittent. The smaller that the proportion of the red globules is in the blood, the more constant this abnormal sound generally is.

It must, however, be admitted, that exceptions are sometimes met with to this assertion; and that, in some rare instances, where there has been no deficiency of the red globules, and no existing organic disease of the orifices of the heart, the blowing sound has been heard.—*Med. Chirurg. Review.* April, 1842.

